# Methylphenidate Enhances Spontaneous Fluctuations in Reward and Cognitive Control Networks in Children With Attention-Deficit/Hyperactivity Disorder

**DOI:** 10.1016/j.bpsc.2022.10.001

**Published:** 2022-10-23

**Authors:** Yoshifumi Mizuno, Weidong Cai, Kaustubh Supekar, Kai Makita, Shinichiro Takiguchi, Timothy J. Silk, Akemi Tomoda, Vinod Menon

**Affiliations:** Department of Psychiatry & Behavioral Sciences, Stanford University School of Medicine, Stanford, California (YM, WC, KS, VM); Research Center for Child Mental Development, University of Fukui, Fukui, Japan (YM, KM, AT); Division of Developmental Higher Brain Functions, United Graduate School of Child Development, University of Fukui, Fukui, Japan (YM, KM, ST, AT); Department of Child and Adolescent Psychological Medicine, University of Fukui Hospital, Fukui, Japan (YM, ST, AT); Department of Neurology and Neurological Sciences, Stanford University, Stanford, California (VM); Wu Tsai Neurosciences Institute, Stanford University, Stanford, California (WC, KS, VM); Maternal & Child Health Research Institute, Stanford University, Stanford, California (WC, KS, VM); Centre for Social and Early Emotional Development and School of Psychology, Deakin University, Geelong, Victoria, Australia (TJS); and Murdoch Children’s Research Institute, Parkville, Victoria, Australia (TJS).

## Abstract

**BACKGROUND:**

Methylphenidate, a first-line treatment for attention-deficit/hyperactivity disorder (ADHD), is thought to influence dopaminergic neurotransmission in the nucleus accumbens (NAc) and its associated brain circuitry, but this hypothesis has yet to be systematically tested.

**METHODS:**

We conducted a randomized, placebo-controlled, double-blind crossover trial including 27 children with ADHD. Children with ADHD were scanned twice with resting-state functional magnetic resonance imaging under methylphenidate and placebo conditions, along with assessment of sustained attention. We examined spontaneous neural activity in the NAc and the salience, frontoparietal, and default mode networks and their links to behavioral changes. Replicability of methylphenidate effects on spontaneous neural activity was examined in a second independent cohort.

**RESULTS:**

Methylphenidate increased spontaneous neural activity in the NAc and the salience and default mode networks. Methylphenidate-induced changes in spontaneous activity patterns in the default mode network were associated with improvements in intraindividual response variability during a sustained attention task. Critically, despite differences in clinical trial protocols and data acquisition parameters, the NAc and the salience and default mode networks showed replicable patterns of methylphenidate-induced changes in spontaneous activity across two independent cohorts.

**CONCLUSIONS:**

We provide reproducible evidence demonstrating that methylphenidate enhances spontaneous neural activity in NAc and cognitive control networks in children with ADHD, resulting in more stable sustained attention. Our findings identified a novel neural mechanism underlying methylphenidate treatment in ADHD to inform the development of clinically useful biomarkers for evaluating treatment outcomes.

Methylphenidate (MPH) is a widely used first-line medication for alleviating clinical symptoms of inattention, hyperactivity, and impulsivity in children with attention-deficit/hyperactivity disorder (ADHD) (1–3). Altered dopamine signaling has been hypothesized to be a key mechanism underlying the therapeutic effects of MPH in ADHD (4). Individuals with ADHD display low dopamine receptor availability in the corticolimbic pathway (5,6), and MPH has been hypothesized to ameliorate ADHD symptoms by increasing extracellular dopamine in the nucleus accumbens (NAc) (7). In neurotypical individuals, dopamine acts as a reinforcer to facilitate motivated behaviors and goal-driven adaptive control (8) via its action on the NAc and cognitive control systems that it regulates (9–12). However, despite decades of its effective use in clinical practice, the precise brain mechanisms underlying the therapeutic effects of MPH are poorly understood, as no consistent findings have emerged to date (13). Specifically, the parallel effects of MPH-induced changes on NAc and its interconnected cognitive control networks and their relation to attentional deficits in childhood ADHD remain unknown.

Dopaminergic pharmacology has been most consistently mapped in the NAc, where dopamine receptors and transporters are particularly dense (7,13). Low dopamine receptor density in the NAc has been linked to the severity of inattention symptoms in adults with ADHD (5). At the brain network level, integrated positron emission tomography (PET)/magnetic resonance imaging (MRI) analyses in neurotypical adults have further revealed that mesolimbic dopamine function influences connectivity of the salience network (SN) and the default mode network (DMN) (14). The SN is important for identifying biologically and cognitively salient events and for guiding attention and goal-directed behaviors (15–18). The SN, the frontoparietal network (FPN), and the DMN constitute a triple-network system (15) that plays a crucial role in a wide range of cognitive tasks that require moment-by-moment changes in adaptive cognitive control (15,19–21). Task-based functional MRI (fMRI) studies of inhibitory control in children with ADHD have suggested that psychostimulants increase activation in the right insula/inferior frontal cortex (22), a key SN node implicated in inhibitory control (16,17). The SN as a locus of deficits in childhood ADHD has been further bolstered by network connectivity analysis of a Go/NoGo task, which identified SN-FPN connectivity as a common locus of deficits in cognitive control and clinical measures of inattention symptoms (23). DMN impairments have also emerged as a prominent feature of ADHD, consistent with theoretical models that have proposed that aberrant engagement of the SN leads to a lack of active suppression and disengagement of the DMN and inattention (24–26). Together, these observations suggest that aberrancies in the NAc together with the SN, FPN, and DMN cognitive control networks may underlie the clinical symptoms of ADHD and constitute specific brain targets for remediation using MPH.

Herein, we used a randomized, placebo-controlled, double-blind crossover design (Figure S1) to investigate the effect of MPH on spontaneous neural activity in the NAc as well as the SN, FPN, and DMN and their links to the behavioral effects of medication in children with ADHD. We used amplitude of low-frequency fluctuation (ALFF) to capture the regional intensity of spontaneous fluctuations in fMRI signals (27). Multimodal PET/MRI studies have suggested that spontaneous fluctuations in fMRI signals arise from metabolic demands associated with ongoing fluctuations in synaptic currents and action potential propagation (28,29). ALFF has been widely used to probe the integrity of brain region–level functioning in psychiatric and neurological disorders (30–33). We used ALFF to test the hypothesis that MPH increases spontaneous neural activity in the NAc, a key node in the dopaminergic reward system and associated cognitive control circuitry.

A critical unaddressed question is whether MPH-induced changes in spontaneous neural activity are related to remediation of attention and cognitive control deficits. Intraindividual response variability (IIRV), a quantitative measure of trialwise performance for behavioral instability, is the most consistent robust behavioral phenotype associated with ADHD (33,34), and psychostimulant treatment reduces this increased variability (33). We recently reported that IIRV in ADHD is associated with poor sustained attention and problems in cognitive control (35). Here, we used a novel similarity metric to measure the extent to which ALFF in the cognitive control network system is similar between children with ADHD and typically developing (TD) children (34). We specifically focused on the triple-network system encompassing the SN, FPN, and DMN in relation to behavioral instability (35) based on extensive evidence for their role in attention and cognitive control (17,21,23,26,36). We hypothesized that children with ADHD, whose postmedication spontaneous activity patterns are more similar to TD children, would exhibit greater improvements in IIRV with medication.

Finally, to address the replication crisis in ADHD (13), we leveraged resting-state fMRI data from a second independent cohort of children with ADHD who participated in a similar randomized controlled trial involving single-dose MPH treatment (37). We tested the hypothesis that multivariate pattern analyses (38) would provide convergent evidence for reproducible findings of MPH-induced changes in spontaneous activity in the NAc and associated cognitive control circuitry in the primary and secondary cohorts.

## METHODS AND MATERIALS

### Participants and Study Design

This study protocol was approved by the Ethics Committee of the University of Fukui, Japan. All participants and their parent(s) provided written informed consent for participation in this study. This study is registered with the University Hospital Medical Information Network (UMIN000027533).

At the University of Fukui Hospital, 34 children with ADHD and 65 TD children were recruited. Figure S1 shows the study design (see Supplemental Methods for details). Children with ADHD were scanned twice in a randomized, placebo-controlled, double-blind crossover design. The administration order was counterbalanced across participants to address potential test-retest issues. During the first visit, they were administered osmotic release oral system (OROS) MPH (1.0 6 0.1 mg/kg) or placebo (lactose) under double-blind conditions as in previous studies (39–41). Five to 8 hours after administration, when the MPH concentration in the blood is maximal (42), the children underwent a resting-state fMRI scan and performed a standardized continuous performance task (CPT) (43,44) outside the MRI scanner.

During the second visit, within 1 to 6 weeks after the first visit, children with ADHD underwent a resting-state fMRI scan and performed the CPT after they took the second medicine: Children who took OROS-MPH at the first visit took the placebo at the second visit under double-blind conditions, and vice versa. The OROS-MPH and the placebo condition are referred to as ADHD-MPH and ADHD-placebo, respectively, in this study.

TD children completed the same resting-state fMRI scan once without either OROS-MPH or placebo. The following inclusion criteria were used for both groups: no contraindications for MRI, Full Scale IQ >70 (to exclude participants with intellectual disability), and no history of severe head trauma or neurological abnormalities (e.g., epilepsy, arachnoid cysts). To minimize the potential impact of sex differences, we included only male participants, consistent with previous ADHD imaging studies (37,45–48). Participants with excessive head motion (>3.0 mm, 3.0°, and mean framewise displacement 0.3 mm) during the scanning were excluded (45). Seven children with ADHD were excluded because of refusal to participate, arachnoid cysts, and motion during the MRI, while 16 TD children were excluded because of psychiatric disorders and neurological abnormalities, leading to a final sample of 27 children with ADHD (mean [SD] age = 10.6 [1.8] years; range, 7.3–15.5 years) and 49 TD control subjects (mean [SD] age = 11.1 [2.3] years; range, 6.1–15.6 years) (Table S1). Of patients with ADHD, 9 had autism spectrum disorder, 6 had oppositional defiant disorder, 2 had specific learning disorder, and 1 had developmental coordination disorder as comorbid disorders. While one of the patients with ADHD was medication-naïve, 25 were medicated with OROS-MPH (mean [SD] medication period = 22.2 [15.3] months; range, 1–58 months), 3 were medicated with atomoxetine, and 2 were medicated with aripiprazole. Children with ADHD took their regularly prescribed medications between the 2 visits, but all participants were medication-free before MRI for at least 5 times half-life, including MPH and atomoxetine, consistent with protocols from previous studies (45,49).

### Assessment of Attention and Cognitive Control

A standardized CPT (43,44) was administered to children with ADHD outside the MRI scanner under both MPH and placebo conditions. The task consisted of a Go/NoGo paradigm in which children were presented with either a target or a nontarget stimulus on the screen for 100 ms, once every 2 seconds for 15 minutes across three 5-minute blocks. The target stimulus was a triangle, while the nontarget stimulus was either a circle or a square. Children were required to press a button when a target stimulus was presented and to withhold response to a nontarget stimulus. The test has been normed to age-adjusted T-scores on 4 distinct performance measures—omission errors, commission errors, mean response time, and IIRV, which was quantified using response time standard deviation (43,44). We examined medication-induced performance differences using paired *t* tests.

### fMRI Data Acquisition

Functional images were acquired with a T2*-weighted gradient-echo echo-planar imaging sequence via a 3T scanner (Discovery MR 750; General Electric Medical Systems) and a 32-channnel head coil. A total of 201 volumes were acquired for a scanning time of 7 minutes 42 seconds. Each volume consisted of 40 slices, with a thickness of 3.5 mm and a 0.5-mm gap. The time interval between each successive acquisition of the same slice (repetition time) was 2300 ms with an echo time of 30 ms and a flip angle of 81°. The field of view was 192 × 192 mm^2^, and the matrix size was 64 × 64, yielding volume dimensions of 3 × 3 mm^3^. The participants were instructed to stay awake with their eyes closed.

### fMRI Data Preprocessing

Resting-state fMRI data were analyzed using SPM12 and DPARSF (50). First, the initial 10 volumes were discarded, and slice-timing correction was performed. The signal from each slice was realigned temporally to that obtained from the middle slice using sinc interpolation. The resliced volumes were normalized to the Montreal Neurological Institute space with a voxel size of 2 × 2 × 2 mm^3^ using the echo-planar imaging template provided by SPM12. The normalized images were spatially smoothed with a 6-mm Gaussian kernel. Next, the non-neural noise in the time series was controlled, and several sources of spurious variance (e.g., the Friston 24-parameter model) were removed from the data through linear regression.

### fMRI Data Analysis

**Figure 1.**
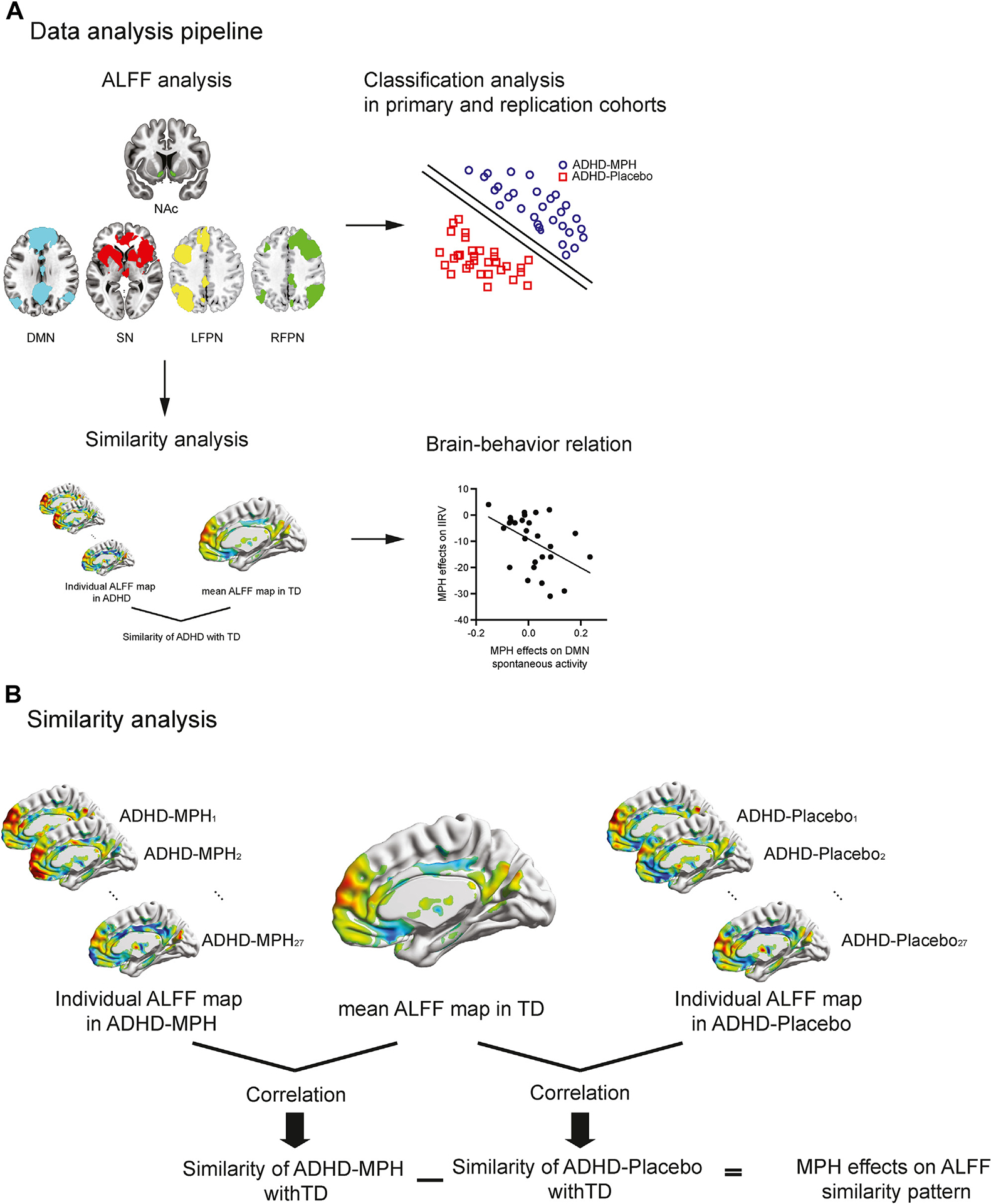
**(A)** Data analysis pipeline. We first computed amplitude of low-frequency fluctuation (ALFF) within the bilateral nucleus accumbens (NAc) and 3 brain networks implicated in attention-deficit/hyperactivity disorder (ADHD): salience network (SN), default mode network (DMN), and left and right frontoparietal network (FPN). Paired *t* tests were used to examine the medication effects (ADHD in methylphenidate [MPH] vs. placebo conditions), and two-sample *t* tests were used to examine the difference between children with ADHD and typically developing (TD) control children. Second, we conducted ALFF pattern similarity analysis **(B)** to quantify the extent to which ALFF values are similar between children with ADHD and TD children and examined whether children with ADHD whose postmedication spontaneous activity patterns were more similar to TD children would exhibit greater improvement in intraindividual response variability (IIRV) with medication. Third, we used classification analysis to test whether the multivariate pattern of ALFF in the NAc and the 3 brain networks could distinguish children with ADHD in medication or placebo conditions (primary cohort) and crucially whether this could be replicated in another independent dataset (replication cohort). **(B)** Overview of ALFF pattern similarity analysis between children with ADHD and TD children. We first computed the correlation between ALFF values within the SN or DMN from each child with ADHD and those from the mean ALFF map in the TD group. The correlation coefficient was standardized using Fisher’s *r*-to-*z* transformation. Next, we calculated MPH-induced changes in the similarity measures of ALFF in the SN or DMN between the conditions of children with ADHD under placebo (ADHD-placebo) and children with ADHD under MPH administration (ADHD-MPH). Higher values indicate that medication leads to more TD-like spontaneous neural activity patterns. LFPN, left FPN; RFPN, right FPN.

Our overall analysis is illustrated in Figure 1A and summarized below (see Supplemental Methods for details).

#### Brain Regions and Networks of Interest.

We focused on the NAc, a key node in the reward pathway, and the SN, DMN, and FPN, 3 core brain systems involved in cognitive control. Probabilistic masks of the bilateral NAc were obtained from an independent high-resolution structural study, and the masks were thresholded at 0.9 (51). The SN, DMN, left FPN, and right FPN maps were obtained from a previous study (24). To test the robustness of our findings, we applied independent component analysis to generate another set of network masks for the SN, DMN, left FPN, and right FPN, using the analytic approach used in our previous study (25).

#### ALFF Analysis.

We assessed spontaneous neural activity by computing the ALFF in bilateral NAc, SN, DMN, left FPN, and right FPN. Paired *t* tests were used to examine the medication effects (ADHD-MPH vs. ADHD-placebo), and two-sample *t* tests were used to examine the difference between children with ADHD and TD children.

#### ALFF Pattern Similarity Analysis.

We evaluated the extent to which ALFF values are similar between children with ADHD and TD children in the SN, DMN, and FPN. We then determined how ALFF similarity is modulated by medication and determined its relationship with medication-induced changes in behavior. We computed an ALFF similarity metric (52) (Figure 1B) using *z*-transformed Pearson’s correlations between ALFF values within each brain network (SN, DMN, or FPN) from each child with ADHD and those from the averaged ALFF map in the TD group. This metric captures the similarity of ALFF patterns in each child with ADHD with respect to the expected patterns in the TD group in each brain region or network of interest. A higher ALFF similarity value indicates that the child with ADHD has a more TD-like ALFF spatial pattern. Medication effect was calculated by subtracting *z*-transformed correlation coefficients in ADHD-placebo from ADHD-MPH conditions. A positive value indicates that medication leads to a more TD-like ALFF spatial pattern. We tested whether medication effects on the ALFF patterns are associated with a behavioral measure of attention and cognitive control, the IIRV, using Pearson’s correlation.

### Replication of MPH Effects on Spontaneous Neural Activity Patterns Using Multivariate Analysis

Finally, we evaluated the replicability of MPH effects on spontaneous neural activity patterns using a second independent cohort of children with ADHD who participated in a similar randomized controlled study involving single-dose MPH treatment. Details of participants and study design are reported elsewhere (37) and summarized in the Supplemental Methods and Table S3. To overcome the limitations of small sample size in the secondary cohort (*n* = 15), we used a multivariate pattern analysis strategy, which facilitates greater reproducibility in comparison to univariate voxelwise measures (38). Specifically, we sought to determine whether MPH would modulate multivariate patterns of ALFF activity in the NAc, SN, DMN, and FPN.

## RESULTS

### MPH Improves Attention and Cognitive Control Function

MPH significantly reduced omission errors, mean response time, and IIRV in the CPT in children with ADHD (all *p* < .001) (see Supplemental Results and Figure S2 for details).

### MPH Effects on Spontaneous Neural Activity in NAc

**Figure 2.**
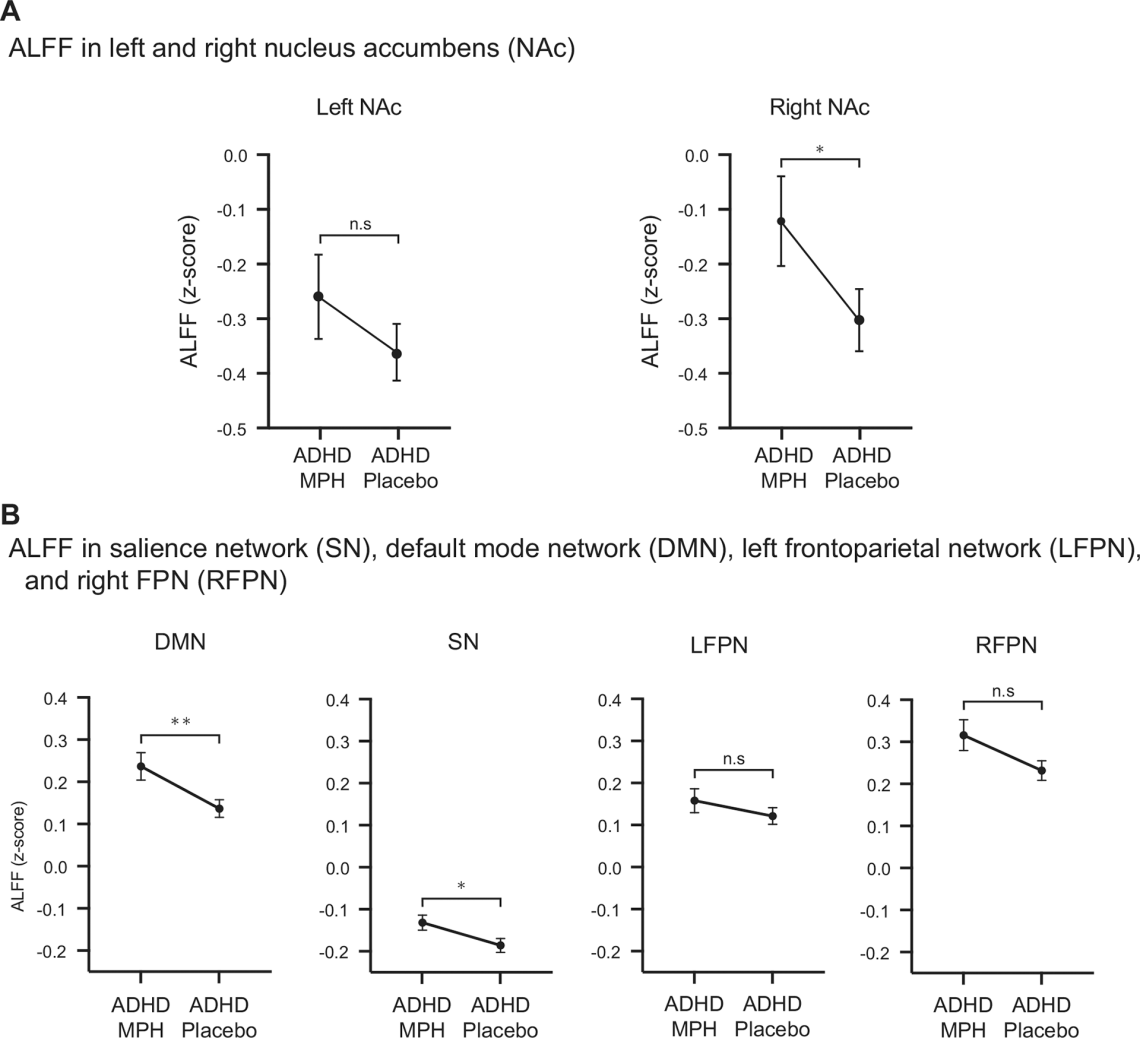
Methylphenidate (MPH) modulated spontaneous neural activity in the nucleus accumbens (NAc) and cognitive control networks. **(A)** MPH increased amplitude of low-frequency fluctuation (ALFF) in the right NAc (*p* < .05, Bonferroni corrected, Cohen’s *d* = 0.43) but not in the left NAc. **(B)** MPH increased ALFF in the default mode network (*p* < .01, Bonferroni corrected, Cohen’s *d* = 0.66) and salience network (*p* < .05, Cohen’s *d* = 0.57) but not in the left and right frontoparietal network. *p < .05; **p < .01. ADHD MPH, children with ADHD under MPH administration; ADHD Placebo, children with ADHD under placebo; n.s, not significant.

ALFF in the right NAc in the ADHD-MPH condition was significantly higher than in the ADHD-placebo condition (*p* < .05, Bonferroni corrected, Cohen’s *d* = 0.55) (Figure 2A) (see Supplemental Results and Figure S3A for comparisons with TD children). These results suggest that MPH enhances spontaneous neural activity in the right NAc.

### MPH Effects on Spontaneous Neural Activity in SN, FPN, and DMN

ALFF in the SN and DMN in the ADHD-MPH condition were significantly higher than in the ADHD-placebo condition (SN: *p* < .05, Bonferroni corrected, Cohen’s *d* = 0.57; DMN: *p* < .01, Bonferroni corrected, Cohen’s *d* = 0.66). There was no significant difference in the left and right FPN (*p* > .05) (Figure 2B) (see Supplemental Results and Figure S3B for comparisons with TD children). Results were replicated using alternate SN, DMN, left FPN, and right FPN masks (see Supplemental Results and Figure S4 for details). These results suggest that MPH enhances spontaneous neural activity in the SN and DMN.

### Relationship Between MPH-Induced Changes in Spontaneous Neural Activity and Changes in Response Variability

**Figure 3.**
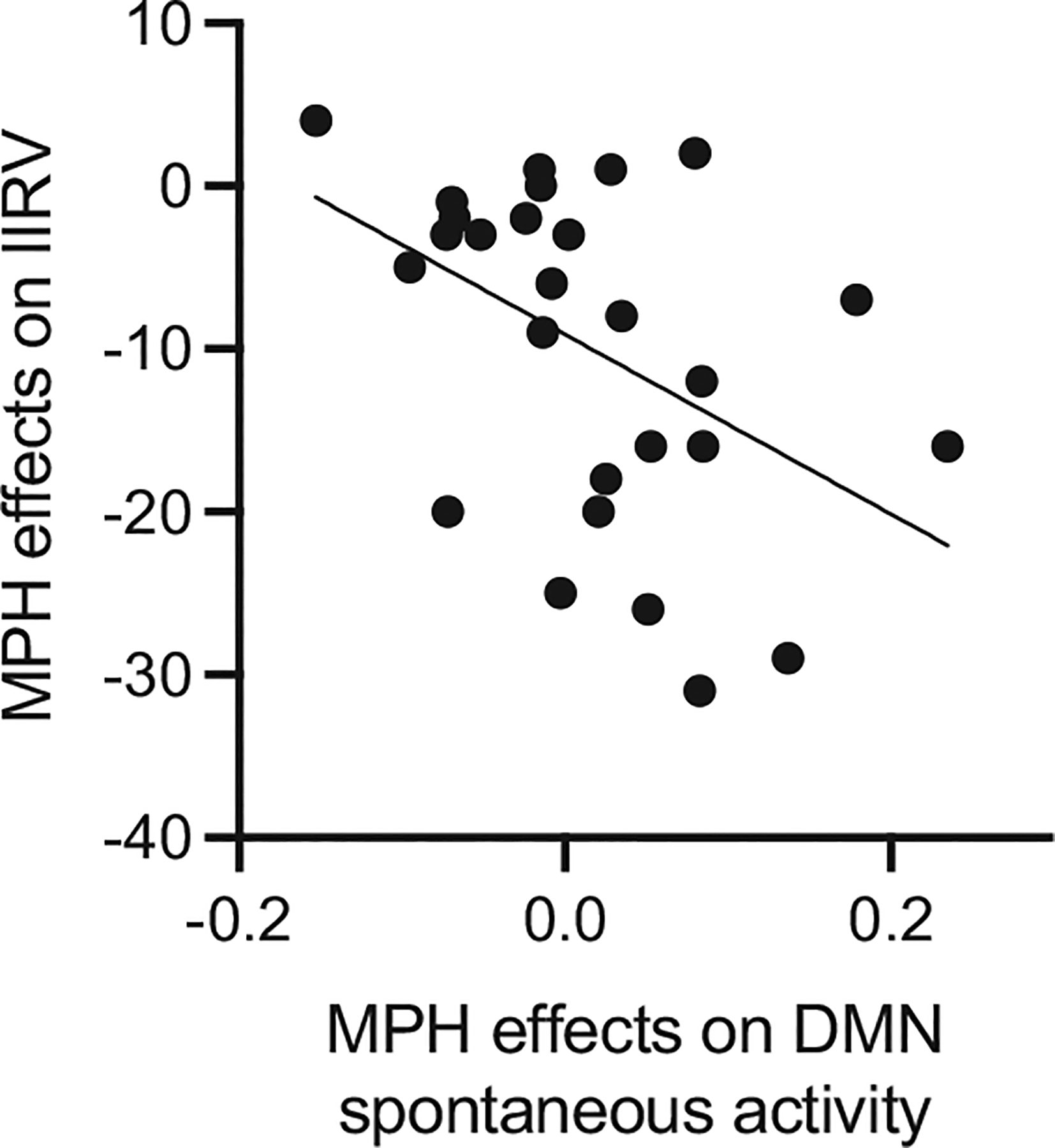
Methylphenidate (MPH) modulation of spontaneous neural activity in the default mode network (DMN) predicted the medication effect on intraindividual response variability (IIRV) (*r* = −20.46, *p* = .016).

We focused on the SN and DMN as these 2 networks showed significant effects of medication on the mean ALFF. We found that medication-induced changes in IIRV were significantly correlated with medication-induced changes in spontaneous activity patterns in the DMN (*r* = −20.46, *p* < .05, Bonferroni corrected) (Figure 3), but not in the SN (*r* = −20.34, *p* = .080). Additional analysis confirmed that the relationship between changes in IIRV and changes in DMN ALFF was robust against several potential confounds (Table 1). Results were replicated using an alternate DMN mask (see Supplemental Results, Figure S5, and Table S2 for details). These results suggest that greater similarity with TD-like ALFF patterns in the DMN after medication is associated with more stable behavioral performance in children with ADHD.

### Replication of MPH Effects on Spontaneous Neural Activity Patterns

**Figure 4.**
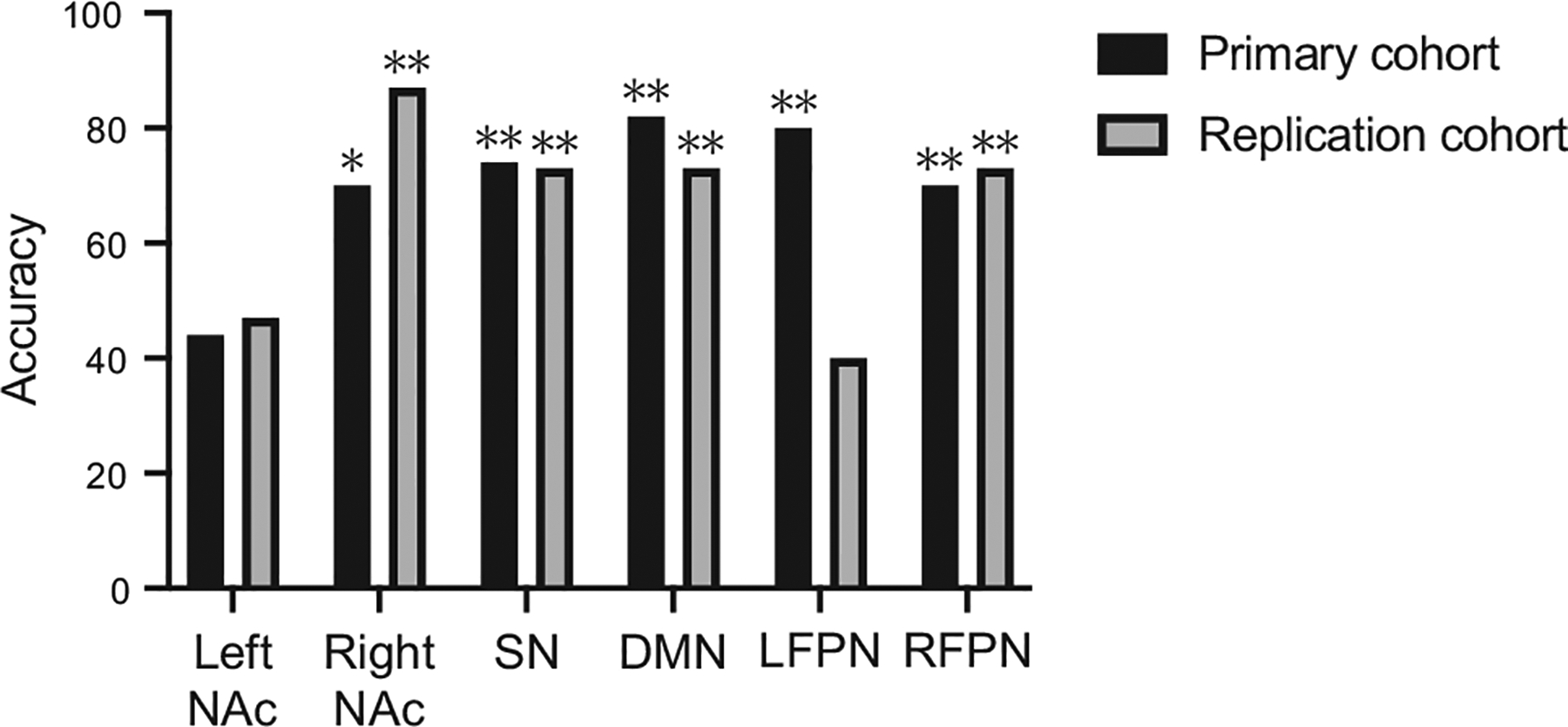
Methylphenidate modulated spontaneous neural activity in the nucleus accumbens (NAc), salience network (SN), default mode network (DMN), and frontoparietal network in children with attention-deficit/hyperactivity disorder: replicable evidence from multivariate classification analyses of primary and replication cohorts. In both the primary and replication cohorts, multivariate patterns of amplitude of low-frequency fluctuation in the right NAc, SN, DMN, and right frontoparietal network (RFPN) distinguished the conditions of children with attention-deficit/hyperactivity disorder under methylphenidate treatment from children with attention-deficit/hyperactivity disorder under placebo. Statistical significance of classification accuracy was estimated using permutation tests. *p < .05; **p < .01. LFPN, left FPN.

Multivariate classification analysis revealed that ALFF differentiated ADHD-MPH and ADHD-placebo conditions in the primary cohort in the right NAc (accuracy = 70%, *p* = .02), SN (accuracy = 74%, *p* = .002), and DMN (accuracy = 82%, *p* = .002). A similar differentiation was observed in the replication cohort (right NAc: accuracy = 87%, *p* = .002; SN: accuracy = 73%, *p* = .002; DMN: accuracy = 73%, *p* = .002) (Figure 4; Table S4). These analyses demonstrate the robustness of our key findings related to MPH-induced changes in spontaneous neural activity patterns in the NAc, SN, and DMN across 2 independent cohorts.

## DISCUSSION

We examined whether MPH alters spontaneous neural activity in the mesolimbic dopaminergic system and cognitive control networks and how these alterations impact cognitive flexibility in children with ADHD. Using a randomized, placebo-controlled, double-blind crossover design, with sample sizes larger than extant randomized controlled studies (13), we showed that MPH alters spontaneous activity in the NAc as well as the SN and DMN, 2 large-scale cognitive control networks implicated in attention and cognitive control deficits in ADHD. Importantly, MPH-induced changes in spontaneous activity patterns in the DMN were associated with improvements in IIRV during a sustained attention task. Finally, in an advance over previous studies, we discovered that MPH alters spontaneous neural activity patterns in the NAc, SN, and DMN and demonstrated replication across 2 independent cohorts of children with ADHD. Together, these findings identify a novel neural mechanism underlying MPH treatment in ADHD.

### MPH Modulates Spontaneous Activity in the NAc

Prominent theories of ADHD have emphasized deficits in the reward and motivation system (53–56). This hypothesis is supported by behavioral findings of aberrant delay discounting, i.e., preference of a small immediate reward over a large delayed reward in children with ADHD and abnormal activation in regions of dopamine reward circuitry during anticipation or processing of rewards in children with ADHD (57,58). As a key node of the dopaminergic reward pathway, the NAc plays an important role in these processes (59,60).

In the present study, we first examined whether MPH alters the spontaneous neural activity of the NAc. We found that compared with placebo, MPH increased spontaneous activity in the NAc in children with ADHD. Our study results converge with PET studies that have reported MPH-induced dopamine increases in the ventral striatum in adults with ADHD (61). Due to the use of radioactive ligands, PET imaging studies cannot be conducted in children. This is an impediment to investigations of MPH-induced dopamine changes in children with ADHD at ages closer to clinical diagnosis, but ALFF measures may offer a useful alternative. In contrast to PET, results with fMRI provide greater anatomical precision and localize MPH-induced effects specifically to the NAc within the ventral striatum. In line with our results, MPH has been reported to increase spontaneous activity in rodent NAc (62), and a recent study in nonhuman primates found that the therapeutic effect of MPH on impulsive decisions is associated with the pharmacological action on the dopamine transporter in the NAc (63). Similarly, in both children and adults with ADHD, MPH has been reported to modify abnormal striatal activity during reward processing (57,64–66). Together, these findings demonstrate that MPH has a strong effect on spontaneous neural activity in the NAc, a key node in the mesolimbic reward pathway, and that the ALFF might be a useful proxy measure to probe MPH effects in children with ADHD.

### MPH Modulates Spontaneous Activity in the SN and DMN

Next, we examined the parallel effects of MPH-induced changes on the SN, FPN, and DMN, 3 large-scale cognitive control networks implicated in ADHD and in attention and cognitive control more broadly (13,16,26,35,52). We found that MPH also increased spontaneous activity in the SN and DMN. Key nodes of the SN, including the anterior insula and anterior cingulate cortex, are among the most highly activated regions in a variety of attention and cognitive control tasks (16,67). Weak activation in the anterior insula and anterior cingulate cortex during cognitive control, especially on error trials, has been reported in children with ADHD (23). Increased attention and cognitive control demand is also accompanied by deactivation in the DMN (21,68,69), and abnormal DMN activity during cognitively demanding tasks is a reproducible feature of ADHD (70). In adults with ADHD, MPH has been shown to increase intrinsic functional connectivity within DMN regions (71) and enhance deactivation of the DMN regions during attentional tasks (72). Our results extend these findings and suggest that one mechanism by which MPH alters cognitive control function is by enhancing spontaneous activity in both the SN and the DMN in children with ADHD.

### MPH Improves Behavioral Performance by Modulating Spontaneous Activity in the DMN

Cognitive control dysfunction is a prominent feature of ADHD, and we recently showed that inattention is correlated with IIRV (35), a key intermediate phenotype of childhood ADHD (73). Several studies have shown that, compared with control subjects, children with ADHD display increased IIRV during cognitive task performance (33,34). We used a novel multivariate pattern similarity measure (52) to determine whether children with ADHD whose spontaneous activity patterns are more similar to TD children after MPH treatment would exhibit greater improvements in IIRV with medication. Our analysis revealed that higher similarity of ALFF patterns in DMN between children with ADHD and TD children was associated with a greater reduction in IIRV in children with ADHD. Previous task-based fMRI studies have reported that activity in the DMN during cognitive performance was related to IIRV and that psychostimulants alter the DMN activity in youths with ADHD (74,75). Our results suggest that the alteration in spontaneous activity in the DMN is a plausible mechanism by which MPH alleviates cognitive inflexibility in children with ADHD. Our results also highlight the specificity of the DMN in terms of its unique association with the effects of medication on IIRV and provide novel evidence that MPH actions on the DMN contribute to remediation of core attention and cognitive control deficits in ADHD (75,76).

### MPH Modulates Multivariate Spontaneous Neural Activity Patterns: Replication Across Two Independent Cohorts

Lack of converging evidence across independent studies is a challenge in clinical neuroscience research, especially in the domain of pharmacological interventions (13). To address this challenge, we sought to replicate key findings in a second cohort of participants from a previously published study (37). Because of the small sample size in the replication cohort (*n* = 15), we used a multivariate pattern analysis approach that has been shown to yield more replicable results than univariate methods (38). Using such an approach, here we report the unprecedented replication of findings in 2 neuroimaging clinical trial cohorts acquired independently. Our analyses revealed that multivariate patterns of spontaneous activity in the NAc as well as in the SN and DMN were modulated by MPH in children with ADHD in both the primary and the secondary cohorts. To the best of our knowledge (13), our replication is the first of its kind and provides confirmatory evidence that MPH alters spontaneous neural activity in key reward and cognitive control systems implicated in childhood ADHD.

### Limitations and Future Work

One limitation of the present study is that fMRI measures cannot establish direct links to changes in dopamine. Future work with hybrid PET/MRI techniques that enable multimodal imaging of different neurotransmitter systems (77) is needed to investigate the impact of MPH on dopamine as well as other neurotransmitters such as norepinephrine and their relationship to spontaneous fluctuations in fMRI signals. Because PET/fMRI studies can be conducted only in adults due to the use of radioactive ligands, the characterization of MPH effects on different neurotransmitter systems in children remains a challenge. As with extant ADHD brain imaging studies, children with ADHD in our study were not drug naïve, were male, and spanned a wide age range from 5 to 16 years. Larger multi-cohort studies that include drug-naïve male and female subjects with ADHD are needed to determine how medication history, sex, and developmental stage modulate MPH effects and to further assess the robustness of the effects reported here.

### Conclusions

Our randomized, placebo-controlled, double-blind crossover study revealed that MPH increases spontaneous brain activity in the reward system at the regional level and in the SN and DMN at the network level. Using a novel ALFF similarity metric, we showed that the effect of MPH on spontaneous activity patterns in the DMN is associated with the effect of medication on IIRV. Strikingly, multivariate analysis demonstrated replicable patterns of MPH-induced changes in spontaneous activity patterns in 2 independent cohorts of children with ADHD. Our findings advance the current understanding of the neurobiological mechanisms underlying MPH treatment in children with ADHD and may lead to clinically useful biomarkers for evaluating treatment response. Finally, our study provides a template for investigations of the effects of MPH on task-related neural activity in striatal reward and related cognitive control circuitry in children with ADHD.

## Supplementary Material

Supplementary Material

Supplementary material cited in this article is available online at https://doi.org/10.1016/j.bpsc.2022.10.001.

## Figures and Tables

**Table 1.** Multiple Linear Regression Analysis Revealed That Only MPH Modulation of ALFF Similarity Pattern Within DMN Was Significantly Associated With Medication Effects on IIRV

Variable	Methylphenidate-Induced Difference in IIRV		
	β	*t*	*p*
MPH Effects on ALFF Similarity Pattern Within DMN	−50.587	−2.333	.029^a^
Age	1.762	1.615	.121
Handedness	−5.553	−0.753	.460
FSIQ	−0.220	−0.970	.342

ALFF, amplitude of low-frequency fluctuation; DMN, default mode network; FSIQ, Full Scale IQ; IIRV, intraindividual response variability; MPH, methylphenidate.

a *p* < .05.
