# Supplementary material for: Methylphenidate Enhances Spontaneous Fluctuations in Reward and Cognitive Control Networks in Children With Attention-Deficit/Hyperactivity Disorder
Source: Biol Psychiatry Cogn Neurosci Neuroimaging. Author manuscript; Available in PMC 2026 Mar 17. (PMC12994527; doi:10.1016/j.bpsc.2022.10.001)
Supplement: Supplementary Material [file NIHMS2150695-supplement-Supplementary_Material.pdf]

SUPPLEMENTAL INFORMATION

Methylphenidate Enhances Spontaneous Fluctuations in Reward and Cognitive Control Networks in Children With Attention-Deficit/Hyperactivity Disorder

Mizuno *et al.*

Contents

I. Supplemental Methods: Primary cohort	p. 2
II. Supplemental Results: Primary cohort	p. 6
III. Supplemental Methods: Replication cohort	p. 7
IV. Supplemental Results: Replication cohort	p. 9
V. Supplemental Figures: Primary cohort	p. 11
VI. Supplemental Tables: Primary cohort	p. 16
VII. Supplemental Tables: Replication cohort	p. 18
VIII. Supplemental Tables: Primary and Replication cohorts	p. 19
IX. Supplemental References	p. 20

I. Supplemental Methods: Primary Cohort

Study design and participants

The overall design of the study is shown in **Figure S1**. 34 children with attention-deficit/hyperactivity disorder (ADHD) were recruited at the University of Fukui Hospital, Japan. Recruitment started in 24/6/2017 and ended in 21/4/2018 because the number of enrolled cases reached the target number. The diagnosis of ADHD was based on the Diagnostic and Statistical Manual of Mental Disorders, Fifth Edition (DSM-5) (1), and was confirmed in structured interviews with investigators using the ADHD module of the Japanese Version of the Kiddie Schedule for Affective Disorders and Schizophrenia for School-Aged Children-Present and Lifetime Version (K-SADS-PL-J)(2,3). Comorbid conditions were evaluated using a semi-structured diagnostic interview, via the Mini International Neuropsychiatric Interview for Children and Adolescents - Japanese version (MINI KID)(4). Additionally, 65 typically developing (TD) children were recruited as control participants from the community between 13/6/2015 and 8/12/2018. The presence of no psychiatric diseases in TD controls was confirmed by MINI KID(4). The TD control group had no family history of psychiatric diseases. Intellectual capacities were estimated via the Wechsler Intelligence Scale for Children-Fourth (WISC-IV)(5). Parents of children in both groups were asked to complete Conners 3rd Edition (Conners) instrument(6) to evaluate inattention, and hyperactivity/impulsivity symptoms under no medication. Handedness was assessed using the Edinburgh Handedness Inventory(7).

Inclusion criteria for both groups were no contraindications for magnetic resonance imaging (MRI), full scale intelligence quotient (FSIQ) > 70 (to exclude participants with intellectual

disability), no history of severe head trauma or neurological abnormalities (e.g., epilepsy, arachnoid cysts). To minimize the potential impact of sex differences we included only male participants, consistent with the male bias in the prevalence of ADHD(8,9). Participants with excessive head motion (over 3.0 mm, 3.0 degree, and mean framewise displacement (FD) 0.3 mm) during the scanning were excluded (10–12).

33 children with ADHD were first randomly assigned to methylphenidate or placebo conditions. One child with ADHD declined to participate and was therefore excluded. The study drug manager assigned the study drug to participants using block randomization. Each time a participant was enrolled, the study drug was assigned according to label of the study drug which was recorded in a random order in advance. During the first visit, participants either took osmotic release oral system methylphenidate (OROS-MPH; 1.0mg/kg: 1.0 ± 0.1mg/kg)(13) or a placebo (lactose) under double-blind conditions. Five to 8 hours after administration of the placebo or methylphenidate(14), when methylphenidate concentration in the blood is maximal(15), participants underwent a resting-state functional MRI (fMRI) scan. Children with ADHD were also administered a continuous performance task (CPT)(16) outside the MRI scanner. Of the 17 children with ADHD who received methylphenidate, 15 completed both MRI and CPT, and 2 were excluded because they did not stay still during the MRI scan. Of 16 children with ADHD who received the placebo, 15 completed MRI and CPT, and 1 was excluded because an arachnoid cyst was detected.

At the second visit, within 1 to 6 weeks from the first visit (mean 17.9 days, standard deviation 9.6 days), they underwent a second resting-state fMRI and performed the CPT again following

administration of either the placebo or methylphenidate under double-blind conditions. Children with ADHD who took OROS-MPH at the first visit now took the placebo at the second visit, and vice versa. All 15 children with ADHD who received methylphenidate completed MRI and CPT, and of the 15 children with ADHD who received the placebo, 12 completed MRI and CPT, and 3 were excluded because 1 declined MRI, and 2 did not stay still during the MRI scan. Adverse events on methylphenidate condition were 2 loss of appetite, 2 insomnia, 1 abdominal pain, and 1 fatigue, all of which were temporal and mild. No serious adverse events were observed. Of 65 TD children, 16 were excluded, as 6 were female, 6 had psychiatric disorders, and 4 had neurological abnormalities. TD controls were scanned once without OROS-MPH or placebo.

Data analyses involved data from 76 subjects, comprising 27 patients with ADHD and 49 TD controls. 14 children with ADHD were classified as combined presentation, and 13 were predominantly inattentive presentation. 9 patients with ADHD had autism spectrum disorder, 6 ADHD patients had oppositional defiant disorder, 2 had specific learning disorder, and 1 had developmental coordination disorder as comorbid disorders. While one of the patients with ADHD was medication-naïve, 25 were medicated with OROS-MPH, three with atomoxetine, and two with aripiprazole. All participants were medication-free prior to MRI for at least 5 times half-lives, including methylphenidate and atomoxetine, consistent with protocol from previous studies(12,17). Mean FD in the ADHD-MPH (0.058±0.014mm) was significantly lower than the ADHD-Placebo and TD groups (*ps* < 0.001, = 0.002). There were no differences in mean FD between the ADHD-Placebo (0.082±0.041mm) and TD groups (0.075±0.033mm) (p = 0.450).

Network identification

To examine generalizability of our findings, we created another set of network masks for SN, FPN and DMN using data from the primary cohort. Preprocessed data from the ADHD and TD samples were concatenated and entered into a group independent component analysis (ICA) to identify large-scale networks in the combined population (MELODIC; http://fsl.fmrib.ox.ac.uk/fsl/fslwiki/MELODIC). The number of components was set to 30, and four components (salience (SN), left and right frontoparietal (FPN), and default mode (DMN) networks) were identified using a quantitative template-matching procedure(18,19). The template matching procedure involved taking the average z score of voxels falling within the template minus the average z score of voxels outside the template and selecting the component in which this difference (the goodness of fit) was the greatest. The templates for SN, DMN, left FPN, and right FPN were identified from previously published studies(20–23). Three investigators (YM, WC, KS) then visually inspected the spatial maps and temporal profiles of each of the 30 ICA components and confirmed the selected SN, DMN, left FPN, and right FPN components.

ALFF analysis

ALFF is a measure of the intensity of spontaneous fluctuations in BOLD signals (24). ALFF was computed at each voxel from the fMRI time series as the square root of power spectrum in the 0.008 to 0.1 Hz frequency range. Voxel-wise ALFF values were standardized into Z-score maps in each individual using the DPARSF toolbox (24–27). Next, mean signals of bilateral NAc and SN, DMN, left FPN, and right FPN from ALFF maps were extracted using the MarsBar toolbox (http://marsbar.sourceforge.net/). Paired *t*-tests were used to examine medication effect (ADHD-

MPH versus ADHD-Placebo) on ALFF in the bilateral NAc, and each network. *p*-values for each network were Bonferroni-corrected for multiple comparisons.

II. Supplemental Results: Primary cohort

Effect of methylphenidate on sustained attention in children with ADHD

To investigate the behavioral consequences of methylphenidate treatment, we examined sustained attention, assessed using the CPT in the ADHD-MPH and ADHD-Placebo conditions. Omission errors, mean RT, and IIRV were significantly lower in the ADHD-MPH, compared to the ADHD-Placebo, condition (all $ps < 0.001$, Cohen's $ds = 0.78, 0.75, 0.97$, respectively; **Figure S2A, S2C, S2D**). There was no significant difference in commission errors between the two conditions ($p > 0.05$, Cohen's $d = 0.03$, **Figure S2B**). These results suggest that a single dose of methylphenidate improves sustained attention deficits in children with ADHD.

ALFF in NAc and in triple network in ADHD-MPH and ADHD-Placebo compared to TD controls

There were no significant differences in ALFF of left and right NAc between ADHD-Placebo and TD controls, and between ADHD-MPH and TD controls ($ps > 0.05$) (**Figure S3A**).

ALFF in DMN, SN, and, LFPN in ADHD-Placebo were significantly lower, compared to TD controls ($ps < 0.001$, < 0.001, < 0.05, Bonferroni corrected, Cohen's $ds = 1.28, 1.17, 0.65$, respectively). However, ALFF in DMN, SN, and LFPN in ADHD-Placebo were not significantly different compared to TD controls ($ps > 0.05$) (**Figure S3B**).

ALFF in salience network (SN), frontoparietal network (FPN), and default mode network (DMN) from independent component analysis under ADHD-MPH and ADHD-Placebo condition

ALFF in SN and DMN was significantly higher in ADHD-MPH (*ps* < 0.05, Bonferroni corrected, Cohen's *ds* = 0.57, 0.52, respectively), compared to ADHD-Placebo conditions, but there were no differences in ALFF in LFPN and RFPN between ADHD-MPH and ADHD-Placebo (*ps* > 0.05) (**Figure S4**).

III. Supplemental Methods: Replication cohort

Study design and participants

To probe the reproducibility of our findings, we analyzed data from a second independent functional neuroimaging cohort involving a randomized controlled study of single-dose methylphenidate in children with ADHD (28). Details of the study design are provided elsewhere (28). Briefly, this dataset consists of 21 patients with ADHD (Age range: 10.0-17.2/Sex: All male). Two participants were excluded because of incompletion of methylphenidate conditions, and 4 were excluded because of excessive head motion (over 3.0 mm, 3.0 degree, and mean framewise displacement (FD) 0.3 mm) during the scanning. As a result, the final sample size was 15 (**Supplemental Table S3**).

Participants were recruited from a specialized clinic at The Royal Children's Hospital, Melbourne defined using the Anxiety Disorders Interview Schedule for Children (A-DISC); a semi-structured diagnostic interview schedule based on DSM-IV criteria. Participants with

ADHD were either medication naïve (n = 9) or withdrawn from medication (n = 6) for at least 48 h prior to the assessment. Comorbidities of opposition deficit disorder and dysthymic disorder were not excluded, whereas pervasive developmental disorders and epilepsy were. All participants had a full-scale IQ >70 according to the WISC-IV.

As part of a randomized, placebo-controlled trial, the effect of methylphenidate was examined with two counterbalanced fMRI sessions separated by a minimum of 2 weeks. One session was done under placebo (lactose) and the other session under an acute dose of methylphenidate (20 mg). Dose (20 mg/participant's weight) ranged from 0.18–0.57 mg/kg with a mean dosage of 0.40 mg/kg (SD = 0.12 mg/kg). MRI scans occurred 1 h 30 mins after administration of the capsules.

fMRI data acquisition

Data were acquired on a 3-Tesla Siemens TIM Trio scanner at The Royal Children's Hospital, Melbourne. Using a 32-channel head coil, participants' heads were secured with foam blocks to restrict movement. T2*-weighted functional images were acquired using a 6.08 min resting-state gradient-echo, echo-planar imaging pulse sequence (repetition time = 2000 ms, echo time = 40 ms, flip angle =90°, field of view =21 cm. Twenty-nine 4.0 mm transverse slices were acquired with an in-plane resolution of 3.0 × 3.0 mm). Participants were instructed to close their eyes and relax. High resolution T1-weighted structural images (TR = 1900 ms, TE = 2.24, flip angle 90, in-plane pixels =0.9 × 0.9 mm) were also collected.

fMRI pre-processing

Resting state fMRI data were preprocessed similarly to primary cohort data. Briefly, first, the initial 10 volumes were discarded, and slice-timing correction was performed. The signal from each slice was realigned temporally to that obtained from the middle slice using sinc interpolation, followed by spatial realignment of 170 volumes to the mean volume. The re-sliced volumes were normalized to the Montreal Neurological Institute space with a voxel size of 2 × 2 × 2 mm using the EPI template provided by SPM12. The normalized images were spatially smoothed with a 6-mm Gaussian kernel. Next, the non-neural noise in the time series was controlled, and several sources of spurious variance (e.g., the Friston 24-parameter model, white matter signals, and cerebrospinal fluid signals) were removed from the data through linear regression.

IV. Supplementary Methods: Primary and Replication cohorts

Multivariate classification analysis of ALFF patterns

We examined whether multivariate ALFF values in the NAc, DMN, SN, left and right FPN can differentiate ADHD-MPH and ADHD-Placebo conditions. We conducted classification analyses using a linear support vector machine (SVM) (C=1) from an open-source library – LIBSVM (https://www.csie.ntu.edu.tw/~cjlin/libsvm/). All voxels in the ALFF map within each ROI or network template were used as features in the classification analysis, and classification accuracy was evaluated using leave-one-out cross validation (LOOCV). Specifically, a random pair of ADHD-MPH and ADHD-Placebo ALFF map from one subject was selected as a test set. The ALFF maps from the rest of the subjects were used to train a classifier to distinguish ADHD-

MPH and ADHD-Placebo group labels. Then the trained classification model was applied to the test set to predict whether the ALFF maps in the test set were ADHD-MPH or ADHD-Placebo. This procedure was repeated N times (N is the number of subjects), where each subject's data used exactly once as a test set. The average prediction accuracy across all test sets is the LOOCV accuracy. Next, a permutation procedure was used to infer statistical significance of LOOCV accuracy. Specifically, in each permutation, data labels (ADHD-MPH vs. ADHD-Placebo) were randomly switched for each subject. LOOCV accuracies from 500 permutations were used to construct the empirical null distribution from which *p*-values for LOOCV accuracies were obtained.

V. Supplemental Figures: Primary cohort

Figure S1. Study design. Randomized placebo-controlled double-blind crossover design to investigate the brain circuit mechanisms that underlie the therapeutic effects of methylphenidate treatment in children with ADHD. CPT: continuous performance task; MRI: magnetic resonance imaging; TD: typically developing.

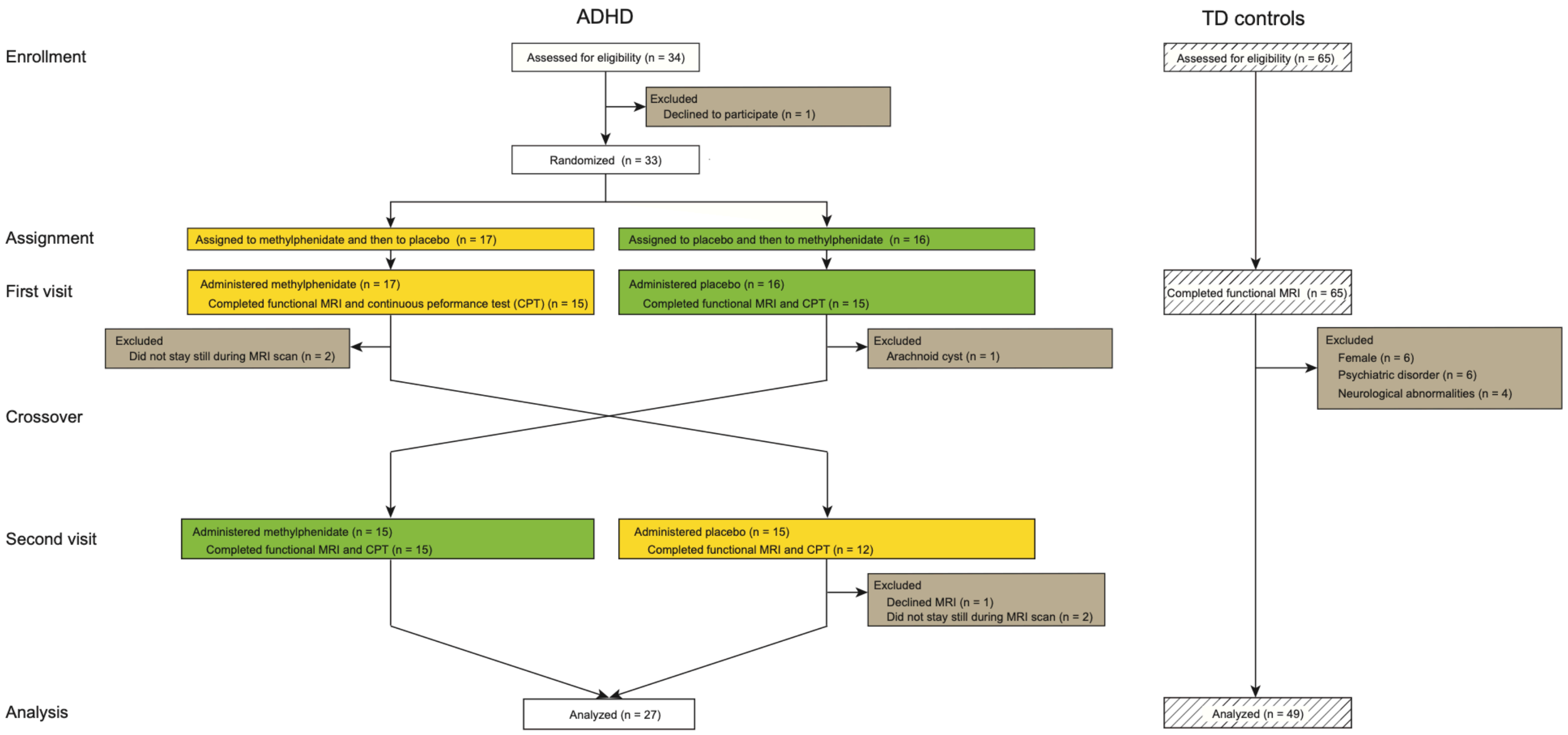

Figure S2. Performance on sustained attention task in children with ADHD under methylphenidate treatment (ADHD-MPH) and placebo condition (ADHD-Placebo). **(A)** Omission errors, **(B)** Commission errors, **(C)** Mean response time, **(D)** Intra-individual response variability (IIRV). Methylphenidate improved omission errors, mean response time, and IIRV. ***p < 0.001; n.s: not significant.

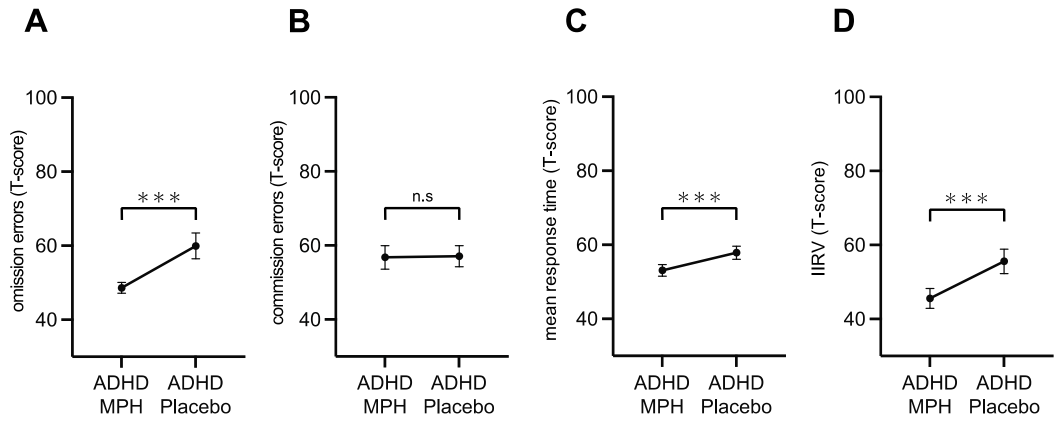

Figure S3. ALFF in left and right nucleus accumbens (NAc) and in salience network (SN), and default mode network (DMN), left frontoparietal network (LFPN), right FPN (RFPN) in children with ADHD under methylphenidate treatment (ADHD-MPH) and placebo condition (ADHD-Placebo) compared to TD controls

***p < 0.001; *p < 0.05; n.s: not significant.

A. ALFF in left and right nucleus accumbens (NAc) in ADHD-MPH and ADHD-Placebo, compared to TD

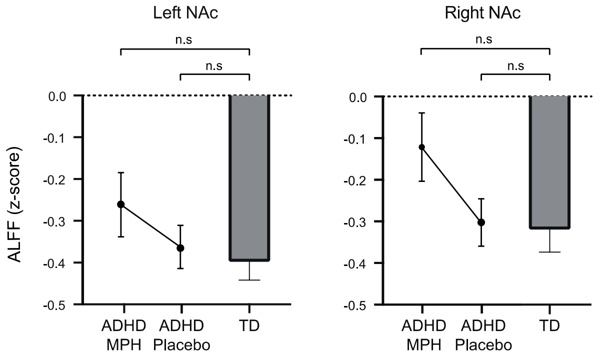

B. ALFF in salience network (SN), default mode network (DMN), left frontoparietal network (LFPN), and right FPN (RFPN) in ADHD-MPH and ADHD-Placebo, compared to TD

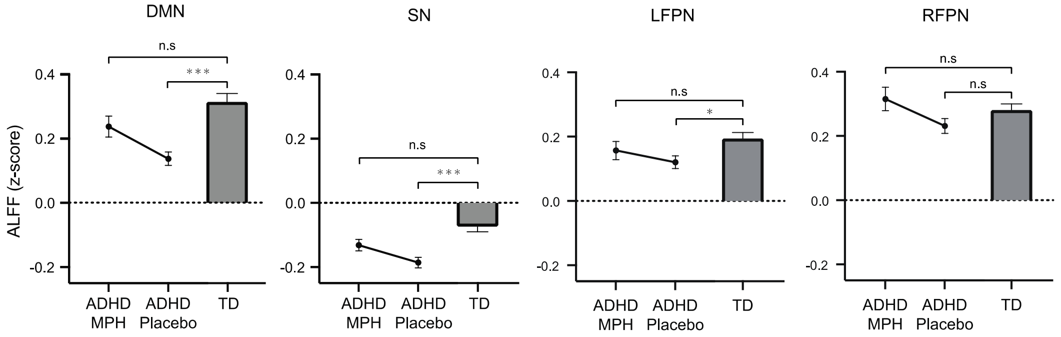

Figure S4. Amplitude of low-frequency fluctuation (ALFF) in salience network (SN), default mode network (DMN), left frontoparietal network (LFPN), and right RFPN (RFPN) from independent component analysis in children with ADHD under methylphenidate treatment (ADHD-MPH) and placebo condition (ADHD-Placebo).

*$p < 0.05$; n.s: not significant.

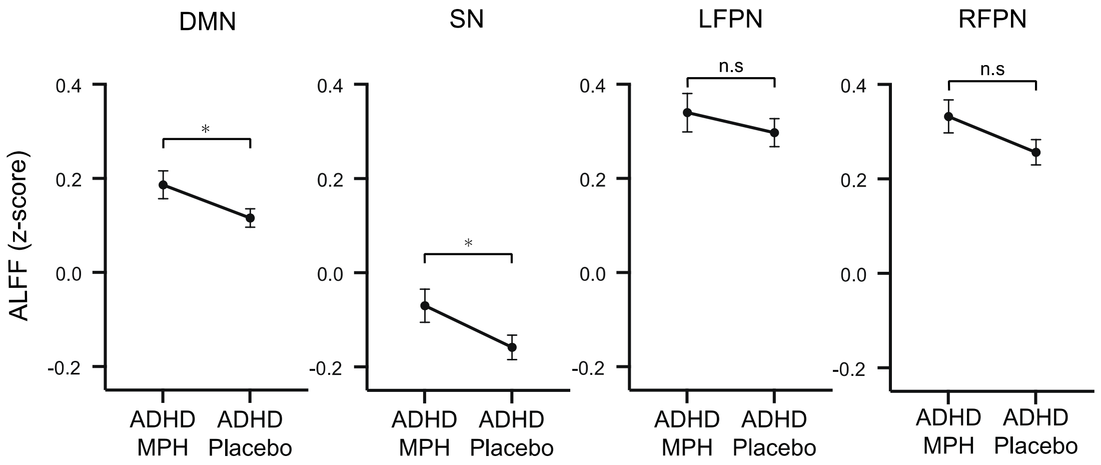

Figure S5. Relation between methylphenidate effects on similarity in ALFF within DMN from independent component analysis and methylphenidate induced difference in IIRV. Medication induced difference in IIRV was significantly correlated with medication effect on similarity in ALFF within DMN (r = -0.46, p = 0.016).

ADHD-MPH: attention-deficit/hyperactivity disorder under methylphenidate treatment; ALFF: amplitude of low-frequency fluctuation; DMN: default mode network; RT: response time; IIRV: intra-individual response variability.

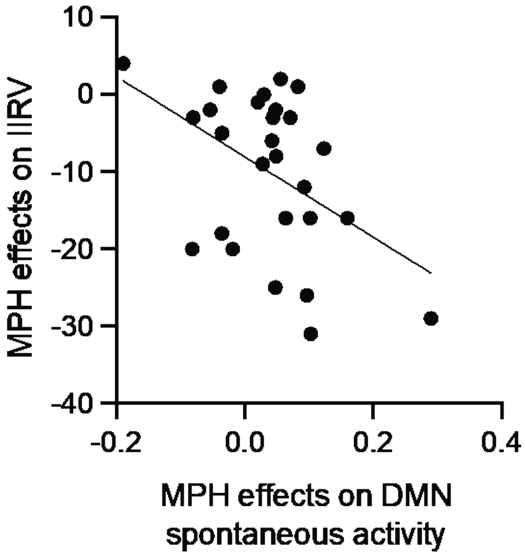

VI. Supplemental Tables: Primary cohort

Supplemental Table S1. Demographic and behavioral characteristics of participants included in data analysis.

	ADHD	TD	*p*
**Sample size**	27	49	-
**Age (years)**	10.6±1.8	11.1±2.3	0.397
**Handedness (R/L)**	25/2	47/2	0.534
**FSIQ**	90.8±8.7	105.2±11.0	<0.001***
**Conners IN (T)**	78.4±12.1	45.7±8.5	<0.001***
**Conners HY (T)**	73.1±15.3	42.9±3.9	<0.001***

ADHD: attention-deficit/hyperactivity disorder; TD: typically developing; R: right; L: left; FSIQ: full scale intelligence quotient; IN: inattention; HY: hyperactivity/impulsivity; ***$p < 0.001$

Supplemental Table S2. Multiple linear regression analysis revealed that only methylphenidate modulation of ALFF similarity pattern within DMN from independent component analysis is significantly associated with medication effects on IIRV.

	Methylphenidate induced difference in IIRV		
	β	***t***	***p***
**Methylphenidate effects on ALFF similarity pattern within DMN**	-46.988	-2.255	0.034*
**Age**	1.670	1.509	0.146
**Handedness**	-6.286	-0.848	0.405
**FSIQ**	-0.203	-0.890	0.383

ALFF: amplitude of low-frequency fluctuation; DMN: default mode network; FSIQ: full scale intelligence quotient; IIRV: intra-individual response variability; $*p < 0.05$.

VII. Supplemental Tables: Replication cohort

Supplemental Table S3. Demographic and behavioral characteristics of participants included in data analysis.

	ADHD
**Sample size**	15
**Age (years)**	13.8±2.4
**Handedness (R/L/B)**	11/2/2
**FSIQ**	94.0±14.9 (n = 14)
**DSM IN (T)**	70.8±6.1
**DSM HY (T)**	75.0±11.8

ADHD: attention-deficit/hyperactivity disorder; TD: typically developing; R: right; L: left; B: both; FSIQ: full scale intelligence quotient; IN: inattention; HY: hyperactivity/impulsivity.

VIII. Supplemental Tables: Primary and Replication cohorts

Supplemental Table S4. Multivariate pattern classification analysis of ALFF in the primary and secondary replication cohorts.

	Primary cohort		Replication cohort	
**ALFF**	Accuracy	*p*	Accuracy	*p*
**Right NAc**	70%	0.020	87%	0.002
**Left NAc**	44%	0.700	47%	0.600
**SN**	74%	0.002	73%	0.002
**DMN**	82%	0.002	73%	0.002
**Right FPN**	70%	0.002	73%	0.002
**Left FPN**	80%	0.002	40%	0.250

ALFF: amplitude of low-frequency fluctuation; DMN: default mode network; NAc: nucleus accumbens; SN: salience network; FPN: frontoparietal network; DMN: default mode network.

IX. Supplemental References

1. American Psychiatric Association (2013): *Diagnostic and Statistical Manual of Mental Disorders: DSM-5*. Washington, DC: American Psychiatric Association. https://doi.org/10.1176/appi.books.9780890425596
2. Kaufman J, Birmaher B, Brent D, Rao U, Flynn C, Moreci P, *et al.* (1997): Schedule for Affective Disorders and Schizophrenia for School-Age Children-Present and Lifetime Version (K-SADS-PL): initial reliability and validity data. *J Am Acad Child Adolesc Psychiatry* 36: 980–8.
3. Miyawaki, D., Suzuki, F., Mamoto, A., Takahashi, K., Kiriike N (2003): The reliability and validity of Japanese version of the schedule for affective disorders and schizophrenia for school-age children — present and lifetime version (K-SADS-PL). *Japanese J Child Adolesc Psychiatry* 197.
4. Sheehan D V, Sheehan KH, Shytle RD, Janavs J, Bannon Y, Rogers JE, *et al.* (2010): Reliability and validity of the Mini International Neuropsychiatric Interview for Children and Adolescents (MINI-KID). *J Clin Psychiatry* 71: 313–326.
5. Wechsler D (2003): *Wechsler Intelligence Scale for Children-WISC-IV*. San Antonio, TX: Psychological Corporation.
6. Conners CK, Pitkanen J, Rzepa SR (2011): Conners 3rd Edition (Conners 3; Conners 2008). In: J. S. Kreutzer, J. DeLuca BC, editor. *Encyclopedia of Clinical Neuropsychology*. New York, NY: Springer New York, pp 675–678.
7. Oldfield RC (1971): The assessment and analysis of handedness: The Edinburgh inventory. *Neuropsychologia* 9: 97–113.
8. Willcutt EG (2012): The prevalence of DSM-IV attention-deficit/hyperactivity disorder: a meta-analytic review. *Neurotherapeutics* 9: 490–499.
9. Xu G, Strathearn L, Liu B, Yang B, Bao W (2018): Twenty-Year Trends in Diagnosed Attention-Deficit/Hyperactivity Disorder Among US Children and Adolescents, 1997-2016. *JAMA Netw open* 1: e181471.
10. Hallquist MN, Hwang K, Luna B (2013): The nuisance of nuisance regression: spectral misspecification in a common approach to resting-state fMRI preprocessing reintroduces noise and obscures functional connectivity. *Neuroimage* 82: 208–25.
11. Zhou Z-W, Fang Y-T, Lan X-Q, Sun L, Cao Q-J, Wang Y-F, *et al.* (2019): Inconsistency in Abnormal Functional Connectivity Across Datasets of ADHD-200 in Children With Attention Deficit Hyperactivity Disorder. *Front psychiatry* 10: 692.
12. Mizuno Y, Jung M, Fujisawa TX, Takiguchi S, Shimada K, Saito DN, *et al.* (2017): Catechol-O-methyltransferase polymorphism is associated with the cortico-cerebellar functional connectivity of executive function in children with attention-deficit/hyperactivity disorder. *Sci Rep* 7: 4850.
13. Wilens T, McBurnett K, Stein M, Lerner M, Spencer T, Wolraich M (2005): ADHD treatment with once-daily OROS methylphenidate: final results from a long-term open-label study. *J Am Acad Child Adolesc Psychiatry* 44: 1015–1023.
14. Bush G, Spencer TJ, Holmes J, Shin LM, Valera EM, Seidman LJ, *et al.* (2008): Functional magnetic resonance imaging of methylphenidate and placebo in attention-

deficit/hyperactivity disorder during the multi-source interference task. *Arch Gen Psychiatry* 65: 102–114.
15. *Concerta® Tablets (Methylphenidate Hydrochloride), Common Technical Document in Japan(October 26 2007、CTD2.7.6.8)* (2007):
16. Huang-Pollock CL, Karalunas SL, Tam H, Moore AN (2012): Evaluating vigilance deficits in ADHD: a meta-analysis of CPT performance. *J Abnorm Psychol* 121: 360–71.
17. Fair DA, Posner J, Nagel BJ, Bathula D, Dias TGC, Mills KL, *et al.* (2010): Atypical default network connectivity in youth with attention-deficit/hyperactivity disorder. *Biol Psychiatry* 68: 1084–1091.
18. Greicius MD, Srivastava G, Reiss AL, Menon V (2004): Default-mode network activity distinguishes Alzheimer's disease from healthy aging: evidence from functional MRI. *Proc Natl Acad Sci U S A* 101: 4637–42.
19. Supekar K, Cai W, Krishnadas R, Palaniyappan L, Menon V (2019): Dysregulated Brain Dynamics in a Triple-Network Saliency Model of Schizophrenia and Its Relation to Psychosis. *Biol Psychiatry* 85: 60–69.
20. Uddin LQ, Supekar KS, Ryali S, Menon V (2011): Dynamic reconfiguration of structural and functional connectivity across core neurocognitive brain networks with development. *J Neurosci* 31: 18578–89.
21. Smith SM, Fox PMTMT, Miller KL, Glahn DC, Fox PMTMT, Mackay CE, *et al.* (2009): Correspondence of the brain's functional architecture during activation and rest. *Proc Natl Acad Sci U S A* 106: 13040–5.
22. Shirer WR, Ryali S, Rykhlevskaia E, Menon V, Greicius MD (2012): Decoding subject-driven cognitive states with whole-brain connectivity patterns. *Cereb Cortex* 22: 158–165.
23. Miller KL, Alfaro-Almagro F, Bangerter NK, Thomas DL, Yacoub E, Xu J, *et al.* (2016): Multimodal population brain imaging in the UK Biobank prospective epidemiological study. *Nat Neurosci* 19: 1523–1536.
24. Zang Y-F, He Y, Zhu C-Z, Cao Q-J, Sui M-Q, Liang M, *et al.* (2007): Altered baseline brain activity in children with ADHD revealed by resting-state functional MRI. *Brain Dev* 29: 83–91.
25. Chao-Gan Y, Yu-Feng Z (2010): DPARSF: A MATLAB Toolbox for "Pipeline" Data Analysis of Resting-State fMRI. *Front Syst Neurosci* 4: 13.
26. Sun Y, Dai Z, Li Y, Sheng C, Li H, Wang X, *et al.* (2016): Subjective Cognitive Decline: Mapping Functional and Structural Brain Changes-A Combined Resting-State Functional and Structural MR Imaging Study. *Radiology* 000: 185–92.
27. Zuo X-N, Di Martino A, Kelly C, Shehzad ZE, Gee DG, Klein DF, *et al.* (2010): The oscillating brain: complex and reliable. *Neuroimage* 49: 1432–1445.
28. Silk TJ, Malpas C, Vance A, Bellgrove MA (2017): The effect of single-dose methylphenidate on resting-state network functional connectivity in ADHD. *Brain Imaging Behav* 11: 1422–1431.
